# HIV-1 variants in population-based national surveys in sub-Saharan Africa, 2015–2022

**DOI:** 10.1097/QAD.0000000000004471

**Published:** 2026-02-16

**Authors:** Peter D. Ghys, Andrew C. Voetsch, Jessica E. Justman, Hetal Patel, Joshua R. DeVos, Kristin Brown, Faith Ussery, Bharat Parekh, Mary Mahy, Alash’le Abimiku, Sani Aliyu, Gambo Aliyu, Khangelani Zuma, Sizulu Moyo, Chéri van der Walt, Monalisa N. Kalimashe, Clement B. Ndongmo, Wilford L. Kirungi, Dawit A. Arimide, Joris Hemelaar

**Affiliations:** a Independent Consultant, Switzerland; b Centers for Disease Control and Prevention, Atlanta, GA; c ICAP at Columbia University, NY, USA; d UNAIDS, Geneva, Switzerland; e Institute if Human Virology, Abuja, Nigeria; f Cambridge University Hospitals, NHS Foundation Trust, Cambridge, UK; formerly AIDS Control Agency; g Formerly AIDS Control Agency, Abuja, Nigeria; h Human Sciences Research Council, Pretoria; i School of Public Health, University of Cape Town, Cape Town; j National Institute for Communicable Diseases, Sandringham, South Africa; k Centers for Disease Control and Prevention, currently at Abidjan, Côte d’Ivoire, formerly at Lusaka, Zambia; l Ministry of Health, Kampala, Uganda; m Department of Translational Medicine, Lund University, Malmö, Sweden; formerly Ethiopian Public Health Institute, Addis Ababa. Ethiopia; n Nuffield Department of Population Health, University of Oxford, Oxford, UK.

**Keywords:** HIV-1, national survey, recombinant, sub-Saharan Africa, subtype

## Abstract

**Objective:**

To describe the distribution of HIV-1 variants in population-based national surveys conducted in sub-Saharan African countries between 2015 and 2022.

**Design:**

Multicountry analysis.

**Methods:**

Sixteen population-based national surveys, conducted between 2015 and 2022, were analyzed. Survey participants were eligible if HIV genotyping was successful. In most countries, people with HIV (PWH) with a recent infection, children with HIV younger than 18 months, and a country-specific selection of PWH with a non-recent infection were included. In Nigeria and South Africa, PWH were eligible when viral load >200 or >1000 RNA copies/ml, respectively. HIV-1 variants were identified using the REGA HIV-1 & 2 Automated Subtyping Tool version 3.0. The estimated distributions of HIV-1 variants for each survey were calculated as the percentage distribution.

**Results:**

The sample size varied between 42 and 1434 PWH per country survey. Country distributions showed great variation, with a majority of CRF02_AG in Cameroon, Côte d’Ivoire and Nigeria; a majority of subtype A in Kenya, Rwanda, and Uganda; near equal proportions of subtype C (39%) and subtype A (37%) in Tanzania, and dominance of subtype C (>90%) in Ethiopia, Eswatini, Lesotho, Malawi, Namibia, South Africa, Zambia, and Zimbabwe. Recombinant viruses were mostly found in countries in West-Africa.

**Conclusions:**

The distribution of HIV-1 variants by country and region in sub-Saharan Africa showed important variation. HIV-1 diversity may need to be accounted for during the development of HIV diagnostic and viral load tests, vaccines, other prevention interventions, and treatment.

## Introduction

The global HIV epidemic remains a global public health problem with 40.8 [37.0–45.6] million people with HIV (PWH), 1.3 [1.0–1.7] million people with a new HIV infection and 630 000 [490 000–820 000] people who died of AIDS in 2024 [1]. Sub-Saharan Africa has historically had the highest HIV prevalence and it remains the most affected region in the world [2].

The extraordinary global genetic diversity of circulating HIV-1 strains forms a major challenge to addressing the HIV epidemic. For HIV-1, four groups have been identified: M, N, O, and P. Among the main (M) group, several subtypes or clades have been described, ranging from subtype A to subtype L, of which subtypes E and I were reclassified as CRF01_AE and CRF04_cpx [3,4]. Recombinant HIV-1 viruses are classified as either circulating recombinant forms (CRFs) or unique recombinant forms (URFs). CRFs are defined as recombinant viruses that are present in three or more epidemiologically unrelated individuals, and as of August 2025, 162 such CRFs were included in the database held by the Los Alamos laboratory [5]. Other recombinant forms are considered URFs [4].

HIV-1 variants are differentially distributed globally and the HIV epidemic is diversifying at the country level, posing challenges to prevention and treatment efforts [6–12]. Of note, approximately one fifth of all HIV-1 infections globally are caused by recombinant HIV strains [12]. Multiple geographical and socioeconomic factors likely contribute to the spread of HIV-1 variants, including transportation networks, migration, founder effects, urbanization, transmission networks, population growth, and availability of treatment and prevention measures [11].

HIV-1 variants may differ in their biological properties, such as rates of transmission and pathogenesis [13]. The performance of diagnostic assays and those for monitoring the response to treatment can vary by HIV-1 variants [14] and vaccine design requires knowledge of local circulating HIV variants [15]. People infected with different HIV-1 variants may also differ in their response to antiretroviral therapy and the development of drug resistance [16].

Up to date and accurate data on the global and national distributions of HIV-1 variants is essential. In the past, HIV-1 variant data (including subtypes or clades, CRFs and URFs) and estimates of their global distribution have typically been informed by surveillance, research studies and programmatic activities [6–12]. As such, they were not representative of the national HIV-1 variant distribution. However, in recent years HIV-1 variant data have become more available with the expansion of genotyping methods of HIV [17] and with the conduct of drug resistance testing in population-based national surveys [18] which are representative of the country's population.

The present study aimed to determine and compare the distribution of HIV-1 variants in people's blood specimens collected through population-based national surveys conducted in sub-Saharan African countries between 2015 and 2022.

## Methods

Population-based HIV impact assessment (PHIA) surveys in the current study were conducted between 2015 and 2022, supported by Columbia University [18]: Cameroon 2017–2018 [19], Côte d’Ivoire 2017–2018 [20], Eswatini 2016–2017 [21], Ethiopia 2017–2018 [22], Kenya 2018–2019 [23], Lesotho 2016–2017 [24], Malawi 2015–2016 [25], Namibia 2017 [26], Rwanda 2018–2019 [27], Tanzania 2016–2017 [28], Uganda 2016–2017 [29], Zambia 2016 [30], and Zimbabwe 2015–2016 [31]. Similar surveys conducted in Nigeria in 2018 [32] and in South-Africa in 2017 [33] and 2022 [34] were also included.

The sampling in the surveys was done in a way that resulted in a sample representative of the national adult population. Procedures of the PHIA surveys have been previously described [35]. In brief, for all surveys, a stratified two-stage cluster sampling technique was used. First, enumeration areas (EAs) were selected with the probability of selection proportional to the size of the EA. Of note, the survey in Ethiopia was conducted in urban areas only. This was followed by the random selection of households from the selected EAs. Finally, all consenting adult (of 15–64 years old generally, in the Tanzania, Eswatini, and South Africa surveys 15+ year old and in the Lesotho and Zambia surveys 15–59 year old) individuals belonging to the households or who had slept in the house the previous night and a proportion of children were eligible for the survey. Consenting individuals (with a dual consent process for children involving the child and a parent/guardian) provided a blood specimen which was tested for HIV using a country-specific rapid test algorithm [36]. For most surveys data collection was performed during <12 months and for all surveys during <20 months.

HIV subtyping was performed in selected laboratories on HIV-positive specimens–after confirmatory HIV testing–which were selected for drug resistance testing and in which amplification of HIV RNA was successful. To avoid replication of proviral DNA, we used RT-PCR which targets viral RNA, thereby avoiding targeting of intracellular pro-viral DNA. The eligibility criteria for HIV-1 subtyping varied by country. In most countries subtyping was conducted on blood specimens from PWH with a recent HIV infection within the prior 12 months–applying a recency algorithm that included the limiting-antigen avidity EIA (LAg), viral load (VL), and the qualitative absence of antiretroviral agents [37] – and all children with HIV younger than 18 months (except for Rwanda for the latter). In addition, a nonrepresentative country-specific selection of PWH with a non-recent infection was also included for most countries. In the two South African surveys, blood specimens from PWH were eligible for subtyping when VL >1000 RNA copies/mL, and in the Nigerian survey PWH when VL >200 RNA copies/ml, regardless of age and the LAg results. The genomic region sequenced was the *pol* gene (protease: 6–99; reverse transcriptase: 1–251) for most surveys and (protease: 6–99; reverse transcriptase: 1–251; and integrase: 1–288) for the second survey in South Africa. The sequencing was done using the Sanger method [38] for most countries and next-generation sequencing (NGS) on the Illumina platform for South Africa. For South Africa, Illumina NGS data were processed using the NGS HIV Drug Resistance pipeline PASeq with a 20% variant frequency threshold to generate a consensus FASTA sequence for subtype analysis. The resulting consensus sequence was then submitted to the REGA HIV-1 subtyping tool, using the same subtype classification algorithm applied to Sanger-derived sequences. Although NGS platforms can detect minority variants below 20%, these low-frequency signals were not incorporated into consensus generation or subtype assignment, and only viral diversity represented at ≥20% was retained in the final sequence. This approach ensures that the dominant viral population is represented and maintains comparability with countries that used Sanger sequencing [39–41]. HIV-1 variants were identified using the REGA HIV-1 and 2 Automated Subtyping Tool version 3.0. Virus variants that were identified as “subtype-like” by the subtyping tool were classified as the relevant subtype.

The number of individuals tested in each survey and the number of HIV-1-positive individuals with viral load results were abstracted from the survey reports [19–34]. Simple proportions were used to describe the proportion of subtyped PWH as the number with a valid subtyping result divided by the number of HIV-1-positive individuals with a valid viral load result. The estimated distributions of variants for each country were calculated as the unweighted percentage distribution of these variants for each survey. The percentage distribution of recombinants other than CRF02_AG was similarly derived.

The survey protocols were approved by the Institutional Review Boards at the U.S. Centers for Disease Control and Prevention (CDC), Columbia University Irving Medical Center, Westat, University of California San Francisco, and University of Maryland, Baltimore and in the respective countries: Comité national d’éthique de la recherche pour la santé humaine (Cameroon); Comité national d’éthique de la recherche (Côte d’Ivoire); Ethiopian public health institute institutional review board (Ethiopia); Health and human research review board (Eswatini); Medical research institute (Kenya); Ministry of Health research and ethics committee (Lesotho); National health sciences research committee (Malawi); Ministry of Health and Social Sciences (Namibia); National health research ethics committee of Nigeria (Nigeria); Rwanda national ethics committee (Rwanda); Human Sciences Research Council (South Africa); National Institute for Medical Research (Tanzania) and Zanzibar Health Research Institute (Zanzibar); Uganda Virus Research Institute and Uganda National Council for Science and Technology (Uganda); Tropical Disease Research Centre and National Health Research Authority (Zambia); and Medical Research Council of Zimbabwe and Research Council of Zimbabwe (Zimbabwe). All participants provided either written or verbal informed consent prior to participating.

## Results

Data were collected from population-based national surveys conducted in the period 2015–2022 in 13 countries that conducted a population-based HIV impact assessment (PHIA), as well as in Nigeria and in South Africa – with two separate surveys in the latter country. A total of 636 593 people were tested for HIV in these national HIV surveys, of whom 38 939 had a valid viral load result (Table 1). The total sample size for this analysis of HIV-1 variants varied between 42 and 1434 PWH per survey, for a total of 4711 PWH. The proportion subtyped varied across surveys, from 2.4% in Zimbabwe to 51.4% in Nigeria. The rate of successful subtyping in the surveys for which we had the relevant information was 74% (out of 1648 samples).

**Table 1:** Characteristics of people with HIV subtyped for HIV-1 in population-based national surveys in sub-Saharan African countries, 2015–2022.

Country	Year of survey	Total number tested for HIV (15–64 year old adults and 0–14 year old children)^*^	PWH with successful viral load measurement	PWH subtyped	Percentage subtyped
West Africa					
Cameroon	2017–2018	33 752	994	81	8.1%
Côte d’Ivoire	2017–2018	32 543	466	91	19.5%
Nigeria	2018	206 210	2790	1434	51.4%
East Africa					
Ethiopia	2017–2018	14 022	631	42	6.7%
Kenya	2018–2019	35 610	1580	103	6.5%
Rwanda	2018–2019	30 637	967	99	10.2%
Tanzania	2016–2017	41 195	1874	255	13.6%
Uganda	2016–2017	39 369	1822	193	10.6%
Southern Africa					
Eswatini	2016–2017	14 306	3099	101	3.3%
Lesotho	2016–2017	15 648	3280	148	4.5%
Malawi	2015–2016	23 353	2316	107	4.6%
Namibia	2017	23 700	2520	150	6.0%
South Africa	2017	23 826	2946	685	23.3%
South Africa	2022	47 683	7652	992	13.0%
Zambia	2016	27 130	2503	146	5.8%
Zimbabwe	2015–2016	27 609	3499	84	2.4%
Total		636 593	38 939	4711	8.3%

* For Rwanda, includes children of 10–14 years only; for Tanzania, Eswatini, and South Africa, includes adults 15 years and older; for Lesotho and Zambia includes adults 15–59 years.

**Fig. 1:**
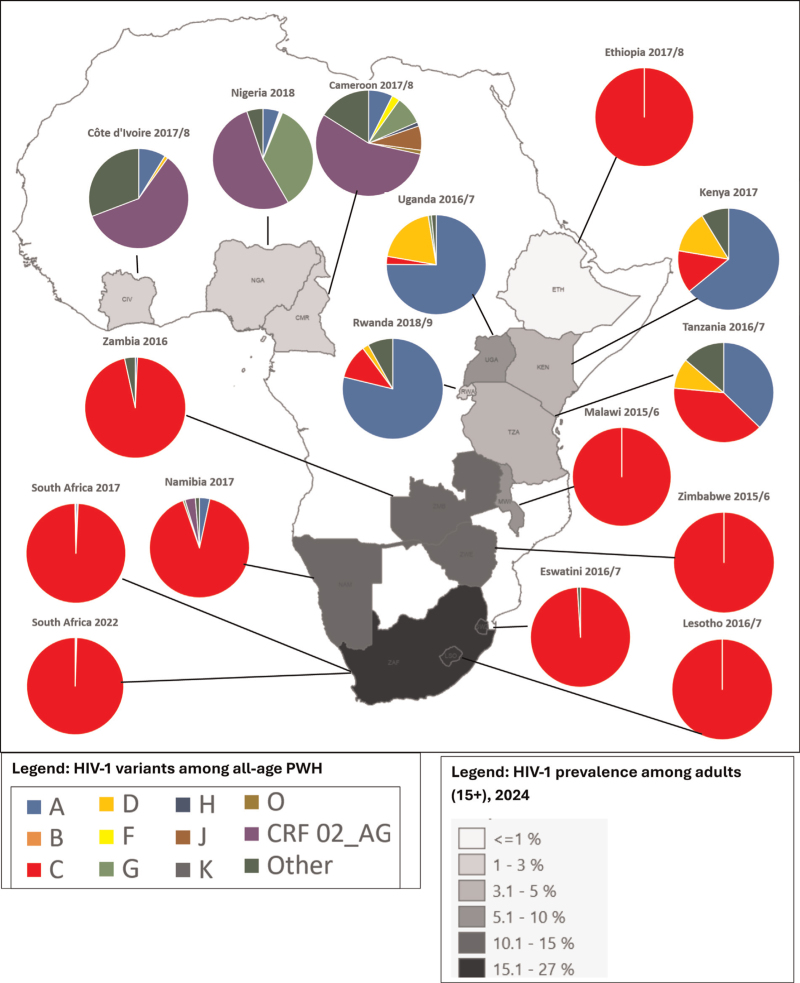
HIV-1 variants in population-based national surveys in sub-Saharan African countries, 2015–2022.

Figure 1 and Table 2 show the country-specific distributions of HIV-1 subtypes, CRF02_AG and other recombinants for each survey. No subtypes K or L were detected. Comparing country distributions showed great variation between countries, with dominance of recombinants (mostly CRF02_AG) in Cameroon, Côte d’Ivoire and Nigeria in West Africa; dominance of subtype A in Kenya, Rwanda and Uganda and near equal proportions of subtype C (39%) and subtype A (37%) in Tanzania in East Africa; and >90% subtype C in Ethiopia, and in Eswatini, Lesotho, Malawi, Namibia, South Africa (both surveys), Zambia and Zimbabwe in southern Africa. The large majority of subtype A viruses for which the sub-subtype was established, were A1 (262/266 = 98.5%), while 1.5% were A2 (note that in Nigeria and Uganda [together harboring 45.5% of all subtype A viruses] the sub-subtype was not specified). The A1 sub-subtype was identified in Cameroon, Côte d’Ivoire, Kenya, Namibia, Rwanda, Tanzania, Zambia, and South-Africa. One sample of Group O was detected in Cameroon. In the Nigeria, Cameroon and Côte d’Ivoire surveys, five or more HIV-1 subtypes and CRFs (10, 9, and 5, respectively) were detected.

**Table 2:** Distribution of HIV-1 variants, sub-Saharan African countries with a national survey, 2015–2022.

Country–year	HIV-1 group M subtypes								HIV-1 group O	Recombinants								Subtotal recombinants			Grand Total
										CRFs^*^						URFs#	Unspecified recombinants	CRFs other than CRF02_AG	Recombinants other than CRF02_AG	All recombinants	
	A	B	C	D	F	G	H	J	O	CRF 02_AG	CRF 06_cpx	CRF 09_cpx	CRF 10_CD	CRF 11_cpx	CRF 37_cpx						
West Africa																					
Cameroon 2017-2018																					
N	6	0	0	0	2	7	1	6	1	45	0	0	0	3	2	7	1	5	13	58	81
%	7.4	0	0	0	2.4	8.6	1.2	7.4	1.2	55.6	0.0	0.0	0.0	3.7	2.5	8.6	1.2	6.2	16.0	71.6	100
Côte d’Ivoire 2017-2018																					
N	8	0	0	1	0	0	0	0	0	54	3	1	0	0	0	21	3	4	28	82	91
%	8.7	0.0	0.0	1.1	0.0	0.0	0.0	0.0	0.0	59.3	3.3	1.1	0.0	0.0	0.0	23.1	3.3	4.4	30.8	90.1	100
Nigeria 2018																					
N	76	4	5	3	3	506	0	0	0	762	56	5	0	6	0	8	0	67	75	837	1434
%	5.3	0.3	0.3	0.2	0.2	35.3	0.0	0.0	0.0	53.1	3.9	0.3	0.0	0.4	0.0	0.6	0.0	4.7	5.2	58.4	100
East Africa																					
Ethiopia 2017–2018																					
N	0	0	42	0	0	0	0	0	0	0	0	0	0	0	0	0	0	0	0	0	42
%	0.0	0.0	100	0.0	0.0	0.0	0.0	0.0	0.0	0.0	0.0	0.0	0.0	0.0	0.0	0.0	0.0	0.0	0.0	0.0	100
Kenya 2018–2019																					
N	66	0	14	14	0	0	0	0	0	0	0	0	0	0	0	5	4	0	9	9	103
%	64.1	0.0	13.6	13.6	0.0	0.0	0.0	0.0	0.0	0.0	0.0	0.0	0.0	0.0	0.0	4.9	3.9	0.0	8.7	8.7	100
Rwanda 2018–2019																					
N	78	0	11	2	0	0	0	0	0	0	0	0	0	0	0	7	1	0	8	8	99
%	78.8	0.0	11.1	2.0	0.0	0.0	0.0	0.0	0.0	0.0	0.0	0.0	0.0	0.0	0.0	7.1	1.0	0.0	8.1	8.1	100
Tanzania 2016–2017																					
N	95	0	100	25	0	0	0	0	0	0	0	0	3	0	0	27	5	3	35	35	255
%	37.3	0.0	39.2	9.8	0.0	0.0	0.0	0.0	0.0	0.0	0.0	0.0	1.2	0.0	0.0	10.6	2.0	1.2	13.7	13.7	100
Uganda 2016–2017																					
N	145	0	5	38	0	2	0	0	0	0	0	0	0	0	0	3	0	0	3	3	193
%	75.1	0.0	2.6	19.7	0.0	1.0	0.0	0.0	0.0	0.0	0.0	0.0	0.0	0.0	0.0	1.6	0.0	0.0	1.6	1.6	100
Southern Africa																					
Eswatini 2016–2017																					
N	0	0	100	0	0	0	0	0	0	0	0	0	0	0	0	1	0	0	1	1	101
%	0.0	0.0	99.0	0.0	0.0	0.0	0.0	0.0	0.0	0.0	0.0	0.0	0.0	0.0	0.0	1.0	0.0	0.0	1.0	1.0.	100
Lesotho 2016–2017																					
N	0	0	148	0	0	0	0	0	0	0	0	0	0	0	0	0	0	0	0	0	148
%	0.0	0.0	100	0.0	0.0	0.0	0.0	0.0	0.0	0.0	0.0	0.0	0.0	0.0	0.0	0.0	0.0	0.0	0.0	0.0	100
Malawi 2015–2016																					
N	0	0	107	0	0	0	0	0	0	0	0	0	0	0	0	0	0	0	0	0	107
%	0.0	0.0	100	0.0	0.0	0.0	0.0	0.0	0.0	0.0	0.0	0.0	0.0	0.0	0.0	0.0	0.0	0.0	0.0	0.0	100
Namibia 2017																					
N	5	0	137	0	0	1	0	0	0	5	0	0	0	0	0	2	0	0	2	2	150
%	3	0.0	91.3	0.0	0.0	0.7	0.0	0.0	0.0	3.3	0.0	0.0	0.0	0.0	0.0	1	0.0	0.0	1.3	1.3	100
South Africa 2017																					
N	4	1	678	0	0	0	0	0	0	0	0	0	0	0	0	2	0	0	2	2	685
%	0.6	0.1	99.0	0.0	0.0	0.0	0.0	0.0	0.0	0.0	0.0	0.0	0.0	0.0	0.0	0.3	0.0	0.0	0.3	0.3	100
South Africa 2022																					
N	4	1	920	0	0	0	0	0	0	0	0	0	0	0	0	0	0	0	0	0	925
%	0.4	0.1	99.5	0.0	0.0	0.0	0.0	0.0	0.0	0.0	0.0	0.0	0.0	0.0	0.0	0.0	0.0	0.0	0.0	0.0	100
Zambia 2016																					
N	1	0	140	0	0	0	0	0	0	0	0	0	0	0	0	5	0	0	5	5	146
%	1	0.0	95.9	0.0	0.0	0.0	0.0	0.0	0.0	0.0	0.0	0.0	0.0	0.0	0.0	3.4	0.0	0.0	3.4	3.4	100
Zimbabwe 2015–2016																					
N	0	0	84	0	0	0	0	0	0	0	0	0	0	0	0	0	0	0	0	0	84
%	0.0	0.0	100	0.0	0.0	0.0	0.0	0.0	0.0	0.0	0.0	0.0	0.0	0.0	0.0	0.0	0.0	0.0	0.0	0.0	100

* CRFs, circulating recombinant forms. ^#^URFs, unique recombinant forms.

**Fig. 2:**
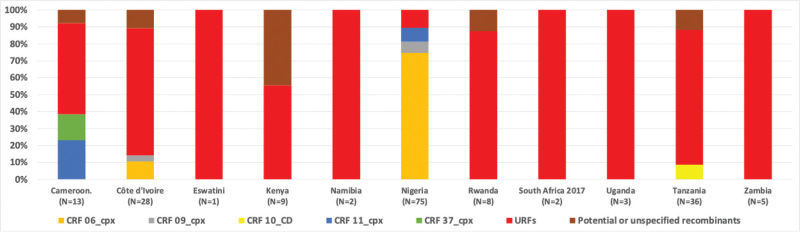
Distribution of recombinant forms other than CRF02_AG, national surveys in sub-Saharan countries, 2015-2022.

The overall proportion of recombinants ranged from 0% in Ethiopia, Lesotho, Malawi, South Africa (2022 survey), and Zimbabwe to 90.1% in Côte d’Ivoire (Fig. 1, Table 2). CRF02_AG was detected in Cameroon (56% of samples), Côte d’Ivoire (59%), Nigeria (53%), and Namibia (3%). Figure 2 shows the country distributions of all recombinants other than CRF02_AG (the latter is included in Fig. 1). CRFs other than CRF02_AG (a total of *n* = 79) were found almost exclusively in West-African countries. They were found in Cameroon (in 6% of samples: three CRF11-cpx and two CRF37_cpx), in Côte d’Ivoire (in 4% of samples: three CRF06_cpx and one CRF09_cpx), in Nigeria (in 5% of samples: fifty-six CRF06_cpx, five CRF09_cpx and six CRF11_cpx) and in Tanzania (in 1% of the samples: three CRF10_CD). Fourteen viruses were classified as unspecified recombinants.

Eighty-eight URFs were identified in the various surveys, slightly more than CRFs other than CRF02_AG. URFs were mostly found in West and East-African countries: Côte d’Ivoire (23% of samples), Tanzania (11%), Cameroon (9%), Rwanda (7%), Kenya (5%), Uganda (2%), Nigeria (1%). None or a very small proportion of URFs was found in Ethiopia (0% of samples) and southern Africa: Eswatini (1%), Lesotho (0%), Malawi (0%), Namibia (1%), South Africa (0.3% in the 2017 survey; 0% in the 2022 survey), Zambia (3%), and Zimbabwe (0%). The identified URFs contained fragments of the following subtypes/CRFs: CRF02_AG/G/A (one sample), CRF02_AG/A (one sample), CRF02_AG/G (19 samples), CRF09_cpx/F (one sample), D/CRF10_CD (one sample), A/C (19 samples), A1/A2 (one sample), A/G (16 samples), A/D (12 samples), B/C (one sample), C/D (10 samples), C/G (one sample), C/H (one sample), C/J (two samples), D/G (one sample) and G/J (one sample).

## Discussion

The population-based distribution of HIV-1 variants in 16 national surveys in sub-Saharan Africa showed important variation, by country and region. In West African countries the recombinant virus CRF02_AG largely dominated the distribution with important proportions of other recombinant viruses (both CRFs and URFs). In countries in East Africa subtype A dominated except for Tanzania where about equal proportions of subtype C and subtype A were detected, with modest proportions of recombinant viruses (mostly URFs) in that region. Finally, in Ethiopia and countries in Southern Africa the distribution was dominated by subtype C, while recombinant viruses were very rare.

A comparison with the most recent analysis based on convenience sampling of surveillance, research studies and programmatic activities at health facilities [12] is not formally possible as that analysis considered regions while the information in the current study is available for specific countries and not all countries in the respective regions are represented. Still in that study, in West-Africa the distribution was dominated by CRF02_AG, in East-Africa subtype A dominated, and in Ethiopia and in southern Africa subtype C dominated, similar to the current study. While the current study does not allow identification of time trends, it shows a great country-to-country variability of recombination, with recombination being especially high in Côte d’Ivoire. Unfortunately, our study did not include data from countries in central Africa.

One sample of group O was identified in Cameroon. Indeed, Cameroon is the country with the highest prevalence of that group [42].

While a decade ago there was no such evidence [43,44], there is recent evidence that sub-subtypes A1/A6 are more likely to develop resistance to cabotegravir [45–47]. In the current study A1 viruses were found in a large number of countries ranging from West- to Southern Africa. As cabotegravir is being rolled out for prevention in these countries, and there is a shared risk for dolutegravir resistance in case of cabotegravir resistance [48,49], the current observation calls for enhanced surveillance and monitoring of resistance and of variants. A study to examine the risk factors for clinical resistance to dolutegravir in patients in participating LMIC countries is being conducted by the IeDEA consortium [50]. Population-based surveys are typically representative of the general population in the country. The exception in the surveys reported here is the Ethiopian survey which included urban and peri-urban areas only. The sampling in most of the surveys reported in this study included PWH with a recent infection, children with HIV and a selection of PWH with a non-recent infection. In the surveys in Nigeria and South Africa eligibility for subtyping blood specimens was determined solely by viral load results and age nor the LAg result were part of the criteria for eligibility. The country-specific distributions of the HIV-1 variants detected in these surveys are representative of the PWH whose blood specimens were tested for antiviral drug resistance. While the selection criteria were different by country, especially for the non-recent infections for all countries but Nigeria and South-Africa, the estimated distributions of HIV-1 variants reported here are more representative of all PWH in each country than the estimated distribution based on convenience sampling, drawing on surveillance, research studies and programmatic activities [6–12].

A limitation of the study is the small sample size for some countries. Indeed, as indicated above and in Fig. 1 and Tables 1 and 2, the smallest absolute sample size was 42 (in Ethiopia) and the smallest percentage of those with a valid viral load measurement was 2.4% (in Zimbabwe). A further limitation is that these results are based on the amplification of a small region of the total genome only. Because of this, the true prevalence of recombination may have been underestimated, especially in countries with a large genetic diversity. Another limitation is the use of different subtyping methods: Sanger in most countries vs. NGS in South Africa. While South Africa used NGS for sequence generation, only variants present at >20% frequency were reported to approximate the ~15–20% detection threshold of Sanger sequencing. This standardized threshold ensured comparability with countries where Sanger sequencing was used and is consistent with expectations for WHO Regional Laboratories to align their outputs with other countries in the WHO HIVDR network. Sanger sequencing predominantly captures only the dominant viral population, so minority HIV variants and mixed infections below this threshold may remain under-represented in countries relying solely on Sanger-based subtyping.

Changing distributions of HIV variants over time need to be monitored to ensure vaccine candidates are designed to match HIV strains circulating in the target populations [51]. Indeed, the Mosaico trial conducted in South-Africa studied a vaccine candidate adapted to the locally dominant subtype C [15].

It is remarkable that one or more variants continue to dominate in certain regions despite substantial migration between countries. For example, it remains unexplained why Ethiopia, located in the East-African region where countries mostly have a subtype A domination, sees a near complete dominance of its (urban) HIV-1 epidemic by subtype C. It has been suggested that it could be related to the absence of major road network connections between Ethiopia and Kenya [52]. A slow shift in variant distribution from subtype D to subtype A has been observed in Rakai, Uganda, according to phylogenetic studies [53]. Furthermore, it has been suggested that subtype C is more virulent and spreads faster than subtypes A and D [54]. This suggests that in sub-Saharan African countries–which have a relatively high prevalence of HIV-1–different transmission or pathogenesis characteristics of HIV-1 variants and a long time period may be needed for changes over time in HIV-1 variant distribution to be observed.

We recommend that future national population-based HIV surveys test for drug resistance and publish the relevant methods and the distribution of HIV-1 variants. In future iterations of the analysis of the global distribution of HIV-1 variants, population-based survey data should be incorporated as a separate stratum with appropriate weighting, in addition to data from convenience sampling drawing on surveillance, research studies and programmatic activities [10–14]. In addition, phylogenetic surveillance of HIV-1 is being expanded in recent years [17] and we recommend that this be accelerated; as a result, more HIV variant data could be available in future years. A formal country-by-country comparison of HIV-1 variant distributions based on samples from national surveys vs. samples from convenience sampling may improve the understanding of the country-specific distribution.

Finally, HIV-1 diversity in sub-Saharan Africa may need to be accounted for during the development of HIV diagnostic and viral load tests as their performance may vary according to the variant distribution [14]. Moreover, as a future vaccine could contribute to ending AIDS as a public health threat, the development of future vaccine candidates should be informed by that diversity [51]. Also, antiretrovirals for prevention and treatment need assessment according to the different HIV-1 subtypes, as some combinations may be prone to the development of resistance [45–50].

## Acknowledgements

We are grateful to all participants in the surveys as well as the staff involved in data collection, testing and analysis. We thank Ian Wanyeki for assistance with Fig. 1.

The Study Group to characterize the HIV-1 variant distribution in national surveys in sub-Saharan Africa, 2015–2022 consists of the following members: Cameroon: Judith Shang, Laura Eno, Alain Arroga Ngotobo, Christophe Mirizoukou; Centers for Disease Control and Prevention, USA: Andrew C Voetsch, Hetal Patel, Joshua R DeVos, Kristin Brown, Faith Ussery, Robert Domaoal, Bharat Parekh; Columbia University; US: Jessica E Justman, Herbert Longwe; consultant: Peter D Ghys; Côte d’Ivoire: Clement B Ndongmo, Christiane Adjé-Touré, Natacha Kohemun, Legre Lobognon, Abo Kouamé; Eswatini: Michelle Adler, Harriet Nuwagaba-Biribonwoha, Sindisiwe Dlamini; Ethiopia: Dan Williams, Yared Tedla Gebrehiwot, Dawit Assefa Arimide; Kenya: Sasi Jonnalagadda, Lily Nyagah, Nancy Bowen; Lesotho: Mugyenyi Asiimwe, Felix Ndagije, Tapiwa Tarumbiswa; Malawi: Nellie Wadonda-Kabondo, Danielle Payne, Rose Nyirenda; Namibia: Leigh Talley, Adam Wolkon, Mike Grasso, Nicholus Mutenda; Nigeria: Alash’le Abimiku, Sani Aliyu, Gambo G Aliyu; Oxford University, UK: Joris Hemelaar; Rwanda: Eugenie Kayirangwa, Sabin Nsanzimana; South Africa: Khangelani Zuma, Sizulu Moyo, Chéri van der Walt, Vibha Kana, Adrian J Puren, Monalisa N Kalimashe, Rindidzani Magobo, Ewaldé Cutler, Leickness Simbayi, Edmore Marinda, Sean Edwin Jooste; Tanzania: Sarah E. Porter, George Mgomella, Optatus Malewo, Prosper Njau; Uganda: Nadia Solehdin, Mary Naluguza, Herbert Kiyingi, Wilford L Kirungi, Sam Biraro; UNAIDS, Switzerland: Mary Mahy; Zambia: Lloyd Mulenga, Nzali Kancheya, Stanley Kamocha, Danielle Barradas; Zimbabwe: Amy Peterson, Priscas Chikwanda, Owen Mugurungi, Raiva Simbi.

Author contributions: P.D.G. and J.H. conceived of the study. P.D.G. collated the data from the national surveys and liaised with representatives of the national surveys (A.A., S.A., G.A., K.Z., S.M., C.V.D.W., M.N.K., C.B.N., W.L.K., and D.A.A. as well as members of the Study Group) to ensure completeness and accuracy of included data. P.D.G. analyzed the data. P.G.D. and J.H. developed tables and figures, interpreted the data, and wrote the first draft of the manuscript.

A.C.V., J.E.J., H.P., K.B., F.U., S.A., G.A., K.Z., S.M., C.B.N., and W.L.K. advised on methodological and sampling aspects of the country surveys.

B.P., J.R.D., A.A., C.V.D.W., and D.A.A. advised on laboratory aspects of the country surveys.

All authors read and approved the final version of the manuscript.

Funding: No specific funding was obtained for this study.

### Conflicts of interest

There are no conflicts of interest.
